# Rapid detection of mutations in the suspected piperaquine resistance gene E415G-exo in *Plasmodium falciparum exonuclease* via AS‒PCR and RAA with CRISPR/Cas12a

**DOI:** 10.1016/j.ijpddr.2024.100568

**Published:** 2024-10-28

**Authors:** Huiyin Zhu, Daiqian Zhu, Yuting Li, Yun Li, Xiaonan Song, Jinyu Mo, Long Liu, Zhixin Liu, Siqi Wang, Yi Yao, He Yan, Kai Wu, Wei Wang, Jianhai Yin, Min Lin, Jian Li

**Affiliations:** a National Institute of Parasitic Diseases, Chinese Center for Disease Control and Prevention (Chinese Center for Tropical Diseases Research), NHC Key Laboratory of Parasite and Vector Biology, WHO Collaborating Centre for Tropical Diseases, National Center for International Research on Tropical Diseases, Ministry of Science and Technology, Shanghai, China; b School of Basic Medical Sciences, Hubei University of Medicine, Shiyan, China; c Department of Pediatrics, Taihe Hospital, Hubei University of Medicine, Shiyan, China; d Wuhan Center for Disease Control and Prevention, Wuhan, China; e Key Laboratory of National Health Commission on Technology for Parasitic Diseases Prevention and Control, Jiangsu Provincial Key Laboratory on Parasites and Vector Control Technology, Jiangsu Institute of Parasitic Diseases, Wuxi, China; f School of Food Engineering and Biotechnology, Hanshan Normal University, Chaozhou, China

**Keywords:** *Plasmodium falciparum*, Single nucleotide polymorphism, *Pfexo* gene, Allele-specific PCR, Recombinase-aided amplification, CRISPR/Cas12a

## Abstract

Malaria remains a major public health concern. The rapid spread of resistance to antimalarial drugs is a major challenge for malaria eradication. Timely and accurate molecular monitoring based on practical detection methods is a critical step toward malaria control and elimination. In this study, two rapid detection techniques, allele-specific PCR (AS**‒**PCR) and recombinase-aided amplification (RAA) combined with CRISPR/Cas12a, were established, optimized and assessed to detect single nucleotide polymorphisms in the *Plasmodium falciparum exonuclease* (*Pfexo*) gene related to suspected piperaquine resistance. Moreover, phosphorothioate and artificial mismatches were introduced into the allele-specific primers for AS**‒**PCR, and crRNA-mismatched bases were introduced into the RAA**‒**CRISPR/Cas12a assay because crRNAs designed according to conventional rules fail to discriminate genotypes. As a result, the detection limits of the AS**‒**PCR and RAA**‒**CRISPR/Cas12a assays were 10^4^ copies/μL and 10^3^ copies/μL, respectively. The detection threshold for dried blood spots was 100**‒**150 parasites/μL, with no cross-reactivity against other genotypes. The average cost of AS**‒**PCR is approximately $1 per test and takes 2**–**3 h, whereas that of the RAA**‒**CRISPR/Cas12a system is approximately $7 per test and takes 1 h or less. Therefore, we provide more options for testing single nucleotide polymorphisms in the *Pfexo* gene, considering economic conditions and the availability of instruments, equipment, and reagents, which can contribute to the molecular monitoring of antimalarial resistance.

## Graphical abstract

**Figure:**
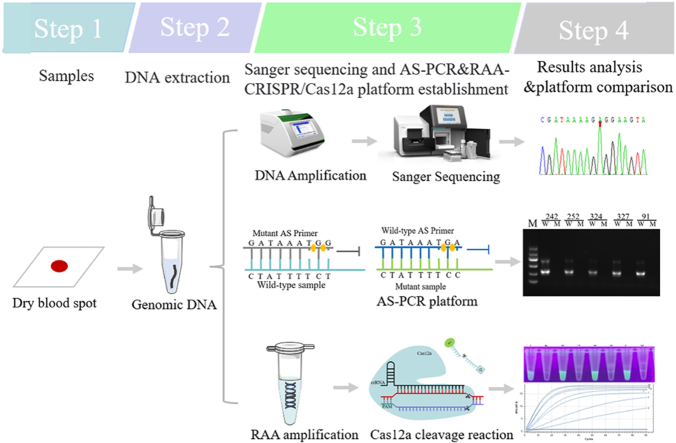

## 1 Introduction

Malaria is a mosquito-borne infectious disease that seriously endangers human health and economic development. There were an estimated 249 million cases of malaria worldwide in 2022, resulting in 608,000 deaths (World Health Organization, 2023). Most deaths and severe cases are caused by *Plasmodium falciparum* (*P. falciparum*). Drug therapy remains the mainstay of malaria prevention and treatment (Okombo et al., 2021; Duffy and Patrick Gorres, 2020). Since June 1998, the World Health Organization (WHO) has recommended the use of artemisinin-based combination therapies (ACTs) as first-line antimalarial therapies; ACTs have saved millions of lives and contributed significantly to the reduction in the global malaria burden over the past two decades (Menard and Dondorp, 2017). However, the recent emergence of artemisinin-resistant *P. falciparum* in Southeast Asia and the spread of resistance to partner drugs have attracted widespread interest. Piperaquine is one of the most common partner drugs in ACTs, and it is safe and effective and has a long half-life (Zongo et al., 2015; PREGACT Study Group et al., 2016). Unfortunately, piperaquine resistance has emerged in several Southeast Asian countries and spread to some African countries, and treatment failures have occurred in both *in vitro* experiments and clinical cases (Amaratunga et al., 2016; Chaorattanakawee et al., 2015; Phuc et al., 2017; Thanh et al., 2017; Phyo et al., 2016). Genome-wide association studies and global allele frequency surveys have revealed that a nonsynonymous mutation (PF3D7_1362500) in an exonuclease gene (exo-E415G) located on chromosome 13 is closely associated with the emergence of piperaquine resistance (Amato et al., 2017; Dondorp, 2017). However, some different views on their relationships still need to be further studied (Boonyalai et al., 2022; Si et al., 2023).

Molecular methods based on nucleic acid amplification are more rapid, sensitive, accurate, and are more effective for detecting antimalarial drug resistance markers than *in vivo* and *in vitro* assays (Zhang et al., 2015). These nucleic acid amplification methods include DNA sequencing and quantitative polymerase chain reaction (qPCR) (Fakhrai-Rad et al., 2002; Cheng et al., 2021; Al-Rumhi et al., 2020). However, most of these methods depend on expensive equipment and well-trained professionals, hindering their clinical application, especially in underdeveloped malaria-endemic countries. Allele-specific PCR (AS**‒**PCR), a new single nucleotide polymorphism (SNP) detection method developed on the basis of the traditional PCR technique, is rapid, sensitive, low-cost, and easy to perform (Barbano et al., 2015; Cheng et al., 2022). Moreover, with the continuous emergence of various infectious diseases, especially after the outbreak of novel coronavirus disease (COVID-19), there is a growing demand for the rapid detection of pathogens using point-of-care testing (POCT) technology. Isothermal amplification techniques are attracting increasing attention, and loop-mediated isothermal amplification (LAMP), recombinase polymerase amplification (RPA), and recombinase-aided amplification (RAA) are the most commonly used methods (Silva Zatti et al., 2020; Liu et al., 2023; Song et al., 2021). In particular, RPA/RAA is an *in vitro* thermostatic (37**–**42°C) nucleic acid amplification technique that use recombinant enzymes, single-stranded binding proteins, and DNA polymerase (Lobato and O'Sullivan, 2018). Additionally, the combination of isothermal amplification technology with the clustered regularly interspaced short palindromic repeats (CRISPR) and associated protein (CRISPR/Cas) system has been successfully developed for detecting several pathogens with increased sensitivity and specificity (Bai et al., 2019; Cunningham et al., 2021; Feng et al., 2021; Wei et al., 2023; Zheng et al., 2024). However, to our knowledge, the use of RAA combined with CRISPR/Cas12a (referred to as RAA**‒**CRISPR/Cas12a) to detect SNPs in drug resistance genes in malaria parasites has not been reported.

In this study, AS**‒**PCR and RAA**‒**CRISPR/Cas12a assays for detecting mutations in the piperaquine resistance gene *Plasmodium falciparum exonuclease* (*Pfexo*) were established, optimized, and evaluated for clinical application. These two technologies can provide more options for simple, rapid, and effective detection of resistance gene mutations.

## 2 Materials and methods

### 2.1 Materials

Taq Plus Master Mix II (2 × ) (Vazyme Biotech Co., Ltd., Nanjing, Jiangsu, China), TIANamp Blood Spot DNA Kit and TIANprep Rapid Mini Plasmid Kit (Tiangen Biotech Co., Ltd., Beijing, China), KOD-Plus-Neo and related PCR reagents (TOYOBO Co., Ltd., Shanghai, China), agarose (Invitrogen, Life Technologies, CA, USA), RAA nucleic acid amplification kits (Hangzhou ZC Bio-Sci & Tech Co., Ltd., Hangzhou, Zhejiang, China), and NEBuffer r2.1, and EnGenR Lba Cas12a (LbCas12a) (New England Biolabs, MA, United States) were used. Most primers were synthesized by Genewiz Biotechnology (Suzhou, Jiangsu, China). RAA primers, ssDNA probes, and crRNA were synthesized by Anyeast Biotechnology Co., Ltd. (Wuhan, Hubei, China).

### 2.2 Sample collection and genotyping

The dried blood spot (DBS) samples used in this study were provided by the Wuhan Center for Disease Control and Prevention, and the DBSs were collected from migrant workers from 2011 to 2019. These patients were first diagnosed via rapid diagnostic kits (Guangzhou Wondfo Biological Co., Ltd.) and then confirmed via microscopic examination of thick and thin blood smears. Moreover, genomic DNA was extracted from DBSs according to the manufacturer's instructions, and the *Pfexo* gene was amplified via traditional PCR (Cavé, 2022). The primers used are listed in Table 1. The reaction was performed in a total volume of 50 μl containing 25 μL of Taq Master Mix II (2 × ), 1.5 μL of each primer (10 μM), 1.5 μL of the DNA template, and 20.5 μL of ultrapure water. PCR was conducted as follows: 1 cycle of 95 °C for 5 min and 40 cycles of 95 °C for 30 s, 53 °C for 30 s,and 72 °C for 10 min on a Bio-Rad T100 Thermal Cycler (Bio-Rad, USA). After the reaction, the PCR products were electrophoresed (100 V, 30 min) on a 1.0% agarose gel. The results were visualized with a UV transilluminator and photographed. The amplified products were purified for Sanger sequencing (Genewiz, Suzhou, China), analyzed with Dnastar (DNASTAR Inc., Madison, WI, USA), and compared with the reference sequence of the *Pfexo* (PF3D7_1362500) gene (https://plasmodb.org/plasmo/app).

**Table 1:** Sequences of primers, crRNA and ssDNA reporter.

Method	Primers	Sequences (5′–3′)	Reference
*Pfexo* molecular surveillance	pfexoF1	TTTCCTTCTGACCCCTTT	[Huang et al., 2015]
	pfexoR1	TCCCATTCGATATCTATACCTAT	
	Sequencing Primer	GGAATGTGCTTTAACGAATGG	
**AS-PCR**	pfexo-W1	CAATATGGTTATAACGATAAA*G*∗G∗A	
	pfexo-W2	CAATATGGTTATAACGATAAA*C*∗G∗A	
	pfexo-W3	CAATATGGTTATAACGATAAA*T*∗G∗A	
	pfexo-M1	CAATATGGTTATAACGATAAA*G*∗G∗**G**	
	pfexo-M2	CAATATGGTTATAACGATAAA*C*∗G∗**G**	
	pfexo-M3	CAATATGGTTATAACGATAAA*T*∗G∗**G**	
	pfexo-R	CGGATTTTCTAATAACCAT	
**RAA**	RAA-F1	CAATAACGATAACGATAATGATAACGATTTAT	
	RAA-F2	ACGATAACGATAATGATAACGATTTATATATG	
	RAA-F3	CGATAATGATAACGATTTATATATGGAATAT	
	RAA-F4	CCTGAAGACGTTAAAAATGTAAAGTACATA	
	RAA-R1	TTATTGTATATATTATTTTCCCAATGATTG	
	RAA-R2	TATAATTTTCATTTGTATAATTAACCATATCC	
	crRNA-Wt1	UAAUUUCUACUAAGUGUAGAUCUUCCUCUUUUAUCGUUAUA	
	crRNA-Mut1	UAAUUUCUACUAAGUGUAGAUCUUCC**C**CUUUUAUCGUUAUA	
	crRNA-Wt2	UAAUUUCUACUAAGUGUAGAUCCAAUGAUUGUUUACUUCCU	
	crRNA-Mut2	UAAUUUCUACUAAGUGUAGAUCCAAUGAUUGUUUACUUCC**C**	
	crRNA-Wt3	UAAUUUCUACUAAGUGUAGAUACAAUAUGGUUAUAACGAUAAAAGA	
	crRNA-Mut3	UAAUUUCUACUAAGUGUAGAUACAAUAUGGUUAUAACGAUAAAAG**G**	
	crRNA-Wt4	UAAUUUCUACUAAGUGUAGAUCUUCCU*G*UUUUAUCGUUAUA	
	crRNA-Mut4	UAAUUUCUACUAAGUGUAGAUCUUCC**C***G*UUUUAUCGUUAUA	
	crRNA-Wt5	UAAUUUCUACUAAGUGUAGAUCUUC*G*UCUUUUAUCGUUAUA	
	crRNA-Mut5	UAAUUUCUACUAAGUGUAGAUCUUC*G***C**CUUUUAUCGUUAUA	
	crRNA-Wt6	UAAUUUCUACUAAGUGUAGAUCUUCCUC*A*UUUAUCGUUAUA	
	crRNA-Wt6	UAAUUUCUACUAAGUGUAGAUCUUCC**C**C*A*UUUAUCGUUAUA	
	ssDNA reporter	5 ′-FAM-TTATT-BHQ1-3′	

Note: Bold letters indicate mutant bases, italics indicate artificially mismatched bases, and ∗ indicates the location of the phosphorothiate modification.

### 2.3 Plasmid construction and identification

The *Pfexo* gene sequence of the *P. falciparum* strain 3D7 was downloaded from PlasmoDB (https://plasmodb.org/plasmo/app). The wild-type and mutant *Pfexo* gene fragments were cloned and inserted into the vector pUC57 to construct recombinant plasmids named *Pfexo* (Wt) and *Pfexo* (Mut), respectively. The recombinant plasmids were extracted from transformed *E. coli* Trans1-T1 phage-resistant chemically competitive cells via the TIAN Prep Mini Plasmid Kit and verified via enzymatic digestion (BamH I and XhoI, Fig. S2A) and sequencing (Fig. S2B). Finally, the concentration and quality were evaluated by Gene5 (Thermo Fisher Scientific, Wilmington, DE, USA), and the number of copies was calculated.

### 2.4 Establishment and application of the AS-PCR system

**Fig. 1:**
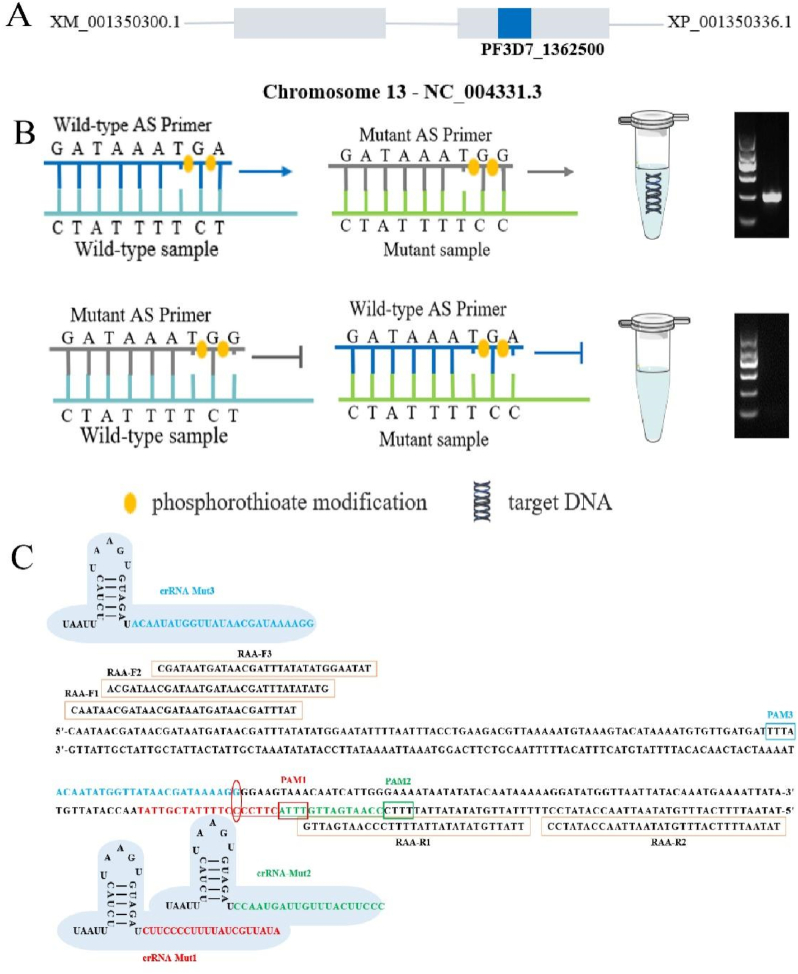
Schematic diagram of the principle of AS‒PCR and RAA‒CRISPR/Cas12a. (A) Schematic diagram showing the location of the *Pfexo* gene. (B) Principle and improvements of AS-PCR, including the introduction of an artificial mismatch and double phosphorothioate modification of the antepenultimate nucleotide at the 3′ end of the AS primer. (C) Positions of RAA primers and crRNAs in the *pfexo* sequence. The red, green and blue boxes are PAMs 1–3, respectively, and the elliptical marker is the SNP site. (For interpretation of the references to colour in this figure legend, the reader is referred to the Web version of this article.)

**Primer design and screening.** Per previous work (Jiang et al., 2021), three candidate primers with different artificial mismatched bases were designed for the antepenultimate bases of the wild-type and mutant allele-specific primers (AS Primers). To improve specificity, we modified the 3′ end of each AS primer by double phosphorothioate modification (Fig. 1). Thus, two AS primers and an artificial reverse primer were designed to distinguish the corresponding alleles (Table 1) (Si et al., 2023).

Wild-type and mutant plasmid DNAs were diluted to 10^9^ copies/μL and used as an amplification templates for cross-amplification with each pair of primers to screen the optimal primers. The PCR system was performed in a 25 μL total volume, including 0.5 U KOD-Plus-Neo, 2.5 μL 10 × buffer, 1.5 μL MgSO_4_ (25 μM), 2.5 μL dNTPs (2 μM), 1 μL each of AS-primer and reverse primer, 1 μL plasmid DNA (pDNA) (10^9^ copies/μL), and 15 μL ddH_2_O. The PCR conditions were as follows: 95 °C for 3 min, 95 °C for 30 s, 58**–**62°C (annealing gradients) for 30 s, 72 °C for 30 s, 30 cycles, and 72 °C for 5 min on a Bio-Rad CFX96 Thermal Cycler (Bio-Rad, USA). Finally, 3 μL of PCR products was analyzed via 1.0% agarose gel electrophoresis (DDY-6D, LIUYI, Beijing, China) at 100 V for 30 min. A primer with a single bright band and no nonspecific amplification was selected as the optimal primer.

**AS‒PCR system optimization**. The amplification efficiency was substantially affected by the primer concentration, MgSO_4_ concentration, annealing temperature, and number of cycles in the reaction system. Accordingly, these parameters were optimized according to the criteria of distinct bands and nonspecific amplification (El-Heliebi et al., 2015). The wild-type and mutant plasmids were used as amplification templates.

**AS‒PCR sensitivity and specificity.** Wild-type and mutant plasmids diluted to 10**‒**10^9^ copies/μL were used as templates, and the wild-type and mutant pDNAs were cross-amplified with wild-type and mutant-type AS primers. The PCR procedure was as follows: 95 °C for 3 min; 95 °C for 30 s, 60.5 °C for 30 s, 72 °C for 30 s, 35 cycles, and 72 °C for 5 min. The specificity was defined as the concentration of the plasmid corresponding to the lightest band visible to the naked eye, and the specificity was evaluated by observing the presence of nonspecific amplification bands.

### 2.5 *Establishment and application of the RAA*‒*CRISPR/Cas12a system*

**RAA primer design and screening.** The primers were designed according to the SNP position of the *Pfexo* gene and RAA primer design rules (Table 1). RAA was performed in a total of 50 μL containing a forward primer and a reverse primer (2.0 μL, 10 μM), 25.0 μL A buffer, 2.5 μL B Buffer, 5.0 μL plasmids or gDNA, 13.5 μL free water and lyophilized enzyme pellets. After 30 min at 39 °C, the amplified products were observed via the CRISPR/Cas12a assay. The entire operation process was conducted in a well-ventilated environment to prevent contamination. After amplification, 3 μL of the amplification product was subjected to electrophoresis (2% agarose gel).

**Design and screening of CRISPR/Cas12a RNA (crRNA).** Wild-type crRNA and mutant crRNA were designed in accordance with the *Pfexo* gene E415G mutation sequence, and an artificial mismatch design was introduced into the crRNA sequence to increase the detection sensitivity. In the CRISPR/Cas12a system, the repeat sequence of "UAAUUUCUACUAAGUGUAGAU" forms the stem-loop structure, and the spacer sequence is complementary to the sequence to be detected. The appropriate protospacer adjacent motif (PAM; TTTN, where N represents any base selected from A, G, C, or T) sequence near the SNP site was identified, where 20**–**25 bp were selected as spacer sequences downstream of the PAM, and T was replaced by U. The sequences are shown in Table 1. The synthesized crRNA was diluted with RNase-free water and stored at **‒**80°C.

**Design of the ssDNA reporter probe.** CRISPR/Cas12a can specifically cleave target dsDNA under the guidance of crRNA and can also indiscriminately cleave nearby nontarget ssDNA. Given this principle, the two ends of the synthetic ssDNA were labeled with fluorescein (FAM) and the fluorescence quenching agent BHQ1 (5′-FAM-TTATT-BHQ1-3′). The Cas12a protein has a better cutting effect on ssDNA sequences rich in bases A and T; thus, the ssDNA sequence of the fluorescent probe was designed as TTATT.

**RAA‒CRISPR/Cas12a system sensitivity and specificity.** Wild-type crRNA and mutant crRNA were cross-reacted with the wild-type and mutant plasmids, respectively, to evaluate the detection specificity. Gradient dilution plasmids (10**‒**10^9^ copies/μL) were used as templates for RAA amplification, and the amplified products were added to the CRISPR/Cas12a reaction system to evaluate the limit of detection (LOD). The CRISPR/Cas12a detection system contained 2.5 μL of 1 μM LbCas12a, 2.5 μL of 10 × NEB buffer, 1.0 μL of 5 μM crRNA, 2.0 μL of 5 μM ssDNA fluorescent reporter, 4.0 μL of RAA products and 13.0 μL of nuclease-free water. The 25.0 μL reaction mixture was then rapidly incubated at 37 °C in a qPCR detection system. The reaction time was set to 45 min, the fluorescence signal value was recorded every 30 s, and the continuous fluorescence value curve was recorded. Finally, the results were observed by the naked eye under an ultraviolet lamp (ZF, KangHua, China) or 485**–**520 nm blue light (LANDUN580, ZEESAN, China).

### 2.6 Evaluation and validation

**Validation for AS‒PCR*.*** To validate the of the optimized AS**‒**PCR system's reliability, 43 clinical *P. falciparum* samples with wild-type *Pfexo* were randomly selected, and five mutant recombinant plasmids were used to simulate mutant samples because of the lack of clinical mutant samples. During detection, each sample to be tested was added to two detection tubes containing wild-type primers or mutant primers respectively, and then amplification, electrophoresis and visualization was carried out. When the amplification band of the sample to be tested appeared in the detection tube containing wild-type primers, the genotype was determined to be the wild type. Similarly, if the band appeared in the detection tube containing the mutant primer, it was determined to be the mutant type. The genotype was classified as mixed type if specific amplification bands were present in both tubes. Finally, the results were compared with the sequencing results for verification.

**Evaluation of the RAA‒CRISPR/Cas12a system.** Two *P. falciparum* clinical samples each with low (≤1000 parasites/μL), medium (1001**‒**10,000 parasites/μL) and high (>10,000 parasites/μL) parasite densities were selected. Since there were no mutant clinical samples, the manually constructed mutant plasmids were used to simulate these samples. Each clinical sample was cross-identified with wild-type crRNA and mutant crRNA, and the genotype identification results were compared with the Sanger sequencing results.

### 2.7 Comparison of these two detection systems

**Fig. 2:**
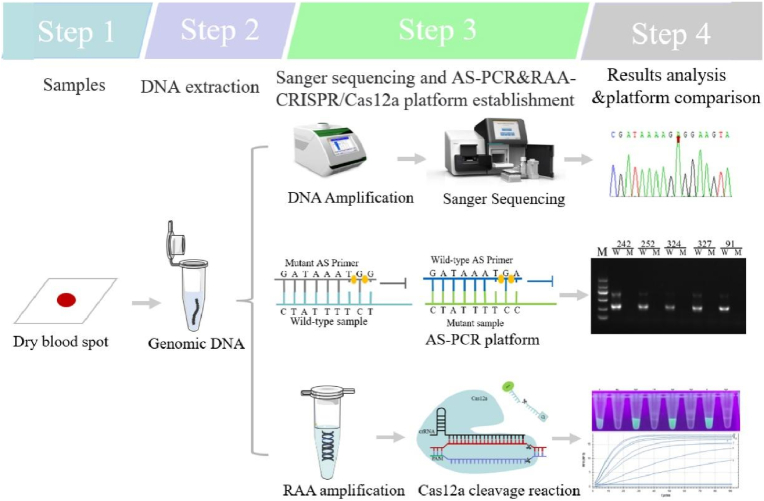
Schematic diagram of this study.

The recombinant plasmid was serially diluted (10**‒**10^9^ copies/μL) to compare the detection limit and specificity of the AS**‒**PCR and RAA**‒**CRISPR/Cas12a systems established in the present study. Moreover, clinical samples with different parasite densities (low, medium, and high) were selected for comparison, and the effectiveness of the two detection systems was evaluated. The detection efficiency, cost and deployment flexibility were also compared (Fig. 2).

## 3 Results

### 3.1 Genetic amplification and genotyping

A total of 309 DBSs from imported *P. falciparum* malaria cases returning from Africa (303) and Southeast Asia (6) to Wuhan were collected from 2011 to 2019. Among the 309 DBS samples, the *Pfexo* gene was amplified (a single band at 618 bp by agarose gel electrophoresis) in 302 samples (Fig. S1A), and 282 (Africa: 276, Southeast Asia: 6) were sequenced successfully. Most cases were from the Democratic Republic of the Congo (15.9%, 44/276), followed by Nigeria (15.6%, 43/276), Angola (10.5%, 29/276), Liberia (8.3%, 23/276), and 26 other countries (49.6%, 137/276). No mutation at the 415 locus of the *Pfexo* gene was detected in the samples that were successfully sequenced; however, three new nonsynonymous mutation sites, D393E (from Liberia), D430Y (from Tanzania), and E437K (from Liberia), were detected in this study (Fig. S1B).

### 3.2 *AS*‒*PCR detection system*

**Fig. 3:**
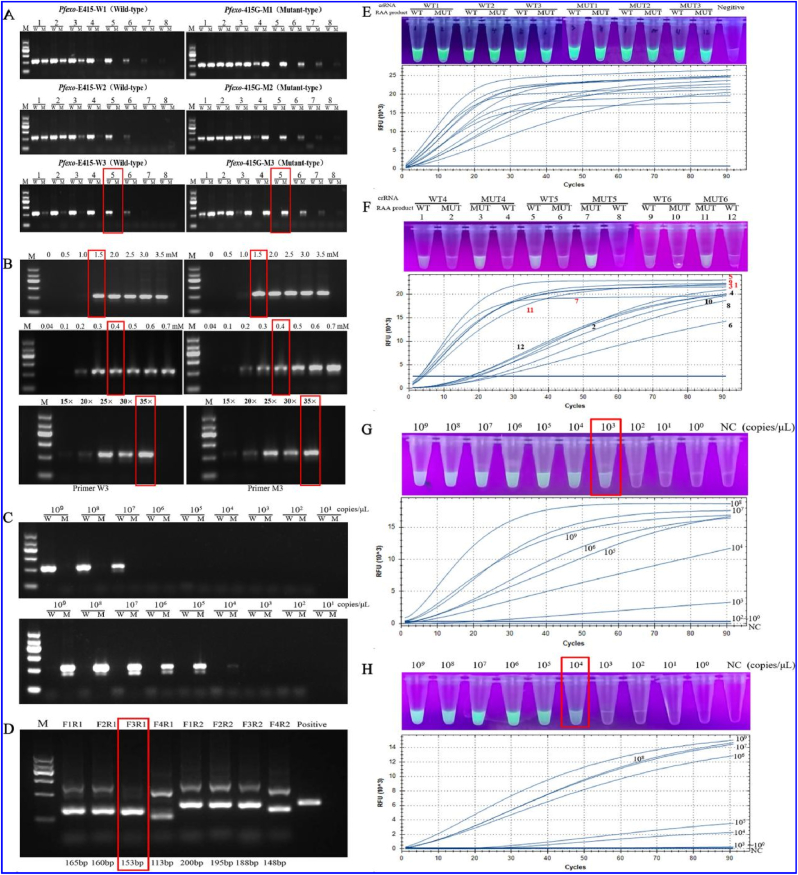
Establishment and optimization of the AS-PCR and RAA-CRSPR/Cas12a platforms. The molecular marker in the electrophoresis agarose gel figures indicating 100 bp, 300 bp, 500 bp, 700 bp, 900 bp and 1200 bp. (A) Primers screening and annealing temperature optimization, 1–8 represent different annealing temperatures of 58.0, 58.3, 58.8, 59.5, 60.5, 61.2, 61.7, and 62.0 °C, respectively. (B) The upper graph shows the optimization of MgSO_4_ concentration; For concentrations of MgSO_4,_ Lane 1–8 were 0, 0.5, 1.0, 1.5, 2.0, 2.5, 3.0 and 3.5 mM, respectively; the middle graph shows the optimization of the concentration of the primers; 1–8 primer concentrations were 0.04, 0.1, 0.2, 0.3, 0.4, 0.5, 0.6 and 0.7 μM; and the lower graph shows the optimization of the number of cycles. 1–5 represent 15, 20, 25, 30 and 35 cycles, respectively. (C) Sensitivity assessment of W3 primer (top panel) and M3 primer (bottom panel). The sensitivity detection is based on wild-type and mutant plasmids as templates, 10^9^-10^1^ copies/μL were plasmid templates at different concentrations. (D) RAA primer screening. (E) Specific detection of crRNA_1∼3_. (F) Specificity detection of crRNA_4∼6_ with mismatch. (G) RAA-CRISPR/Cas12a LOD on wild-type samples. (H) RAA-CRISPR/Cas12a LOD on mutant-type samples.

For the selection of wild-type primers (Fig. 3A), the amplification efficiencies of the three candidate AS primers, W1, W2, and W3, were similar, and all demonstrated false positives when the mutant template was used at an annealing temperature lower than 58.8 °C. As the annealing temperature gradually increased, W3 manifested a clear single band earliest and was selected as the most specific primer. Similarly, M3 was also selected as the most specific AS primer for the mutant primers.

For optimization of the AS**‒**PCR system, AS**‒**PCR annealing temperatures were set with the following gradients: 58.0, 58.3, 58.8, 59.5, 60.5, 61.2, 61.7, and 62.0 °C (Fig. 3A). For the wild-type primers or mutant primers, a single band of the expected size (328 bp) was observed. For the selected wild-type primer *Pfexo*-W3, the number of amplification bands gradually decreased as the annealing temperature increased to 61.7 °C. Moreover, nonspecific amplification occurred when the annealing temperature was below 58.8 °C. Conversely, the band was bright and single when the annealing temperature was 60.5 °C. Consequently, the most effective annealing temperature of the *Pfexo*-W3 primer was 60.5 °C. Similarly, 60.5 °C was selected as the appropriate annealing temperature for *Pfexo*-M3.

The concentration of Mg^2+^ in the PCR system affects the amplification efficiency and specificity. In this study, the final concentration of MgSO_4_ in the reaction system was set as the following gradient: 0, 0.5, 1.0, 1.5, 2.0, 2.5, 3.0, and 3.5 mM (Fig. 3B). The bands could not be detected when the MgSO_4_ concentration was lower than 1.0 mM; the bands gradually became brighter as the MgSO_4_ concentration increased, and the bands were clear and bright when the MgSO_4_ concentration in the reaction system was 1.5 mM. Thus, 1.5 mM was selected as the MgSO_4_ concentration for the subsequent experiments.

The concentration of each primer was subsequently optimized. The primer concentrations were set to 0.04, 0.1, 0.2, 0.3, 0.4, 0.5, 0.6, and 0.7 mM. With increasing primer concentration, the target bands gradually became brighter, and the bands were bright and single when the concentration increased to 0.4 mM (Fig. 3B). Accordingly, a primer concentration of 0.4 mM was selected as the final concentration for both the wild- and mutant-type primers.

The number of cycles was optimized using the findings above. Single bands were observed after 15, 20, 25, 30, and 35 cycles (Fig. 3B). Finally, 35 was adopted as the optimal cycle number for AS**‒**PCR.

In addition, the constructed wild-type and mutant recombinant plasmids were diluted in a tenfold gradient (10**‒**10^9^ copies/μL) to simulate different loads of *P. falciparum* infection (Fig. 3C). The results revealed that the optimized AS**‒**PCR system had a low detection limit of 10^4^ copies/μL (27.4 fg/μL) for the *Pfexo* mutant gene. There was no cross-amplification between the mutant primers and the wild-type plasmid, indicating high sensitivity and specificity.

### 3.3 RAA-CRISPR/Cas12a system

First, the *Pfexo* (Wt) and *Pfexo* (Mut) plasmids were used as templates for RAA amplification. As shown in Fig. 3D, all *Pfexo* gene target fragments were successfully amplified. The F3R1 primer was selected as the best RAA primer because the target band was bright, and there was no nonspecific amplification.

Second, wild-type crRNA (crRNA-Wt1**‒**3) and mutant crRNA (crRNA-Mut1**‒**3) were cross-reacted with wild-type and mutant RAA amplification products, respectively. As shown in Fig. 3E, when the RAA amplification product was genotypically identical to the crRNA, the ssDNA probe was cleaved, the fluorescence signal appeared in the tube under test, and the relative fluorescence unit (RFU) exceeded 15 × 10^3^. However, when the two genotypes were inconsistent, they could not be distinguished. Therefore, artificially mismatched bases were introduced into wild-type crRNA (crRNA-Wt4**‒**6) and mutant crRNA (crRNA-Mut4∼6) (Fig. 3F) and then cross-reacted with the RAA products again. The results illustrateed that when the RAA amplification product and crRNA genotypes were consistent, the ssDNA probe was successfully cleaved, and the tube to be tested showed a fluorescence signal with an RFU greater than 20 × 10^3^. When the RAA amplification product and crRNA genotypes were inconsistent, there was no obvious fluorescence signal visible to the naked eye in the tube to be tested within 20 cycles (10 min reaction), and the shape of the fluorescence curve clearly differed from that when the genotypes were the same. The most significant difference in fluorescence values was found between crRNA-Wt5 and crRNA-Mut5; consequently, they were used in the system.

Third, tenfold gradient dilutions (10-10^9^ copies/μL) of the wild-type and mutant plasmids were used as templates for RAA amplification, and the amplified products were added to the CRISPR/Cas12a system for genotype identification. As shown in Fig. 3G and H, for the detection of the wild-type and mutant plasmids, fluorescence signals were observed in the test tube when the template concentration was above 10^3^ copies/μL and 10^4^ copies/μL, respectively. The RFU curve was generated via RT-qPCR. Accordingly, the RAA**‒**CRISPR/Cas12a system's detection limits for the for the wild-type and mutant templates were 10^3^ and 10^4^ copies/μL, respectively. Additionally, a high degree of specificity was observed, with the wild-type template being undetectable by the mutant crRNA and the mutant template being undetectable by the wild-type crRNA.

### 3.4 Clinical evaluation

**Fig. 4:**
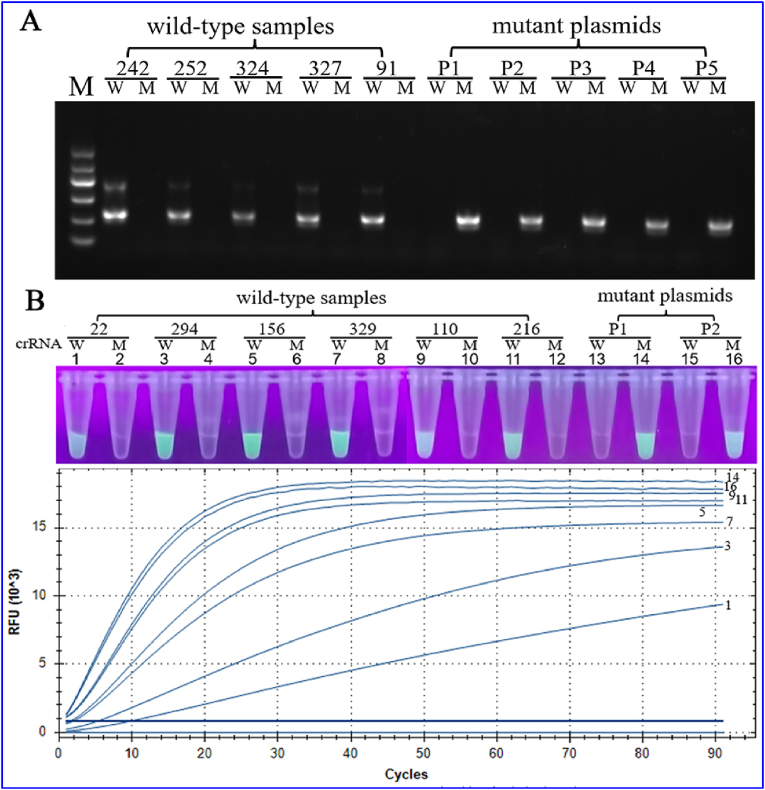
Clinical isolate assessment of the AS-PCR and RAA-CRSPR/Cas12a platforms. (A) Agarose gel electrophoresis of some clinical isolates detected by AS-PCR platform, 42, 252, 324, 327 and 91 represent the numbers of wild-type clinical isolates, and their parasite densities were 500, 500, 3,000, 5000 and 28,000 parasites/μL, respectively. P1-P5 represent mutant plasmids. The molecular marker in the electrophoresis agarose gel figures indicating 100 bp, 300 bp, 500 bp, 700 bp, 900 bp and 1200 bp. (B) Some clinical isolates detected by RAA-CRISPR/Cas12a platform. 22, 294, 156, 329, 110 and 216 represent the numbers of wild-type clinical isolates, and their parasite densities were 150, 1,000, 7,000, 5,000, 500,000 and 85,000 parasites/μL, respectively; 1–16 represent sample tube numbers.

Forty-three wild-type clinical samples were selected for the clinical validation of AS**‒**PCR, comprising 16 from patients with low parasitemia, 16 from those with medium parasitemia and 11 from those with high parasitemia. For the *Pfexo*-E415G mutation, the wild-type and mutant types were successfully distinguished, 43 wild-type clinical samples were successfully amplified via wild-type primers, five mutant recombinant plasmids were successfully amplified via mutant primers, nonspecific amplification was not performed, and the target bands were obtained via electrophoresis (Fig. 4A). All the results were consistent with the Sanger sequencing results. The LOD was 100 parasites/μL, and no false-positive or false-negative results were found (Table 2). The AS**‒**PCR testing system requires an average of $1 for a single test.

**Table 2:** Results of the clinical application of AS-PCR.

Genotype and parasitemia (parasites/μL)	Seqencing No.	AS-PCR		Sensitivity (%)	Specificity (%)	False negative (%)	False positive (%)
		WT	Mut				
Wild-type clinical samples							
Low parasite density (≤1000)	16	16	0	100	100	0	0
Medium parasite density (1001-10,000)	16	16	0	100	100	0	0
High parasite density (>10,000)	11	11	0	100	100	0	0
Mutant plasmids	5	0	5	100	100	0	0
Total	48	43	5	100	100	0	0

For clinical validation of the RAA**‒**CRISPR/Cas12a system, two samples each were selected from the low, medium, and high density samples, and mutant plasmids were used to replace mutant clinical samples. Each clinical sample was cross-identified with wild-type and mutant crRNA. Finally, the system correctly identified clinical samples with different parasite densities; the lowest identified parasite density was 150 parasites/μL (Fig. 4B), and an average of $7 for a single test was estimated.

### 3.5 Comparison of these two detection systems

For the wild-type samples, the detection limits of AS‒PCR and RAA‒CRISPR/Cas12a were 10^7^ copies/μL and 10^3^ copies/μL, respectively, and for the mutant samples, they all had an LOD of 10^4^ copies/μL (Table 3). For clinical sample validation, both systems demonstrated consistency with Sanger sequencing. For detection efficiency, starting from the completion of sample DNA extraction, the AS‒PCR assay takes 2–3 h, whereas RAA‒CRISPR/Cas12a takes 1 h or less. Regarding reagent cost, AS‒PCR and RAA‒CRISPR/Cas12a cost approximately $1.0 and $7.0 per test, respectively. To obtain genotyping results, AS‒PCR first requires electrophoresis and then by UV gel imaging equipment, whereas the genotype identificantion results of RAA‒CRISPR/Cas12a can be observe by the naked eye through irradiating the test tube with blue light.

**Table 3:** Characteristics of AS-PCR and RAA-CRISPR/Cas12a systems.

	AS-PCR	RAA-CRISPR/Cas12a
Limit of detection	10^4^ copies/μL	10^3^ copies/μL
Clinical specificity	100%	100%
Detection period (not including extraction time)	2∼3 h	Result within 1 h
Reagent cost	$1	$7
Dependence on equipment	Rely on PCR equipment, electrophoresis and visualization equipment	Low equipment dependence, results can be viewed by the naked eye under blue light
Advantages	Lower test cost, require fewer types of reagents	Higher detection sensitivity and efficiency, low equipment dependence, relatively simple operation, more suitable for field detection
Disadvantages	Complex operation process, more time consuming	Need more expensive reagents, Such as, RAA amplification reagent, Cas12a enzyme

## 4 Discussion

With the widespread use of ACTs as a first-line treatment for *P. falciparum* malaria, malaria incidence and mortality have decreased significantly in the past 20 years (Bhatt et al., 2015). However, the emergence and rapid spread of resistance to artemisinins and their partner drugs are major obstacles to malaria control and elimination (Ashley et al., 2014; Huang et al., 2015). Therefore, continuous monitoring of imported malaria and its drug resistance is important for malaria elimination efforts. This study established two methods, AS‒PCR and RAA‒CRISPR/Cas12a, to adapt to different scenarios for detecting SNPs in the *P. falciparum* drug resistance gene *Pfexo*. These methods demonstrated high sensitivity and specificity and cost-effectiveness.

The E415G mutation in the *Pfexo* gene is associated with the failure of piperaquine (Witkowski et al., 2017) and is one of the best candidate SNP markers of piperaquine resistance to help monitor the spread of these phenotypes (Amato et al., 2017). One study revealed that 93% (13/14) of the patients who experienced treatment failure in Vietnam in 2015 carried the piperaquine resistance marker exo-E415G mutation (Thanh et al., 2017). However, a study conducted in Senegal using 76 *P. falciparum* isolates showed that approximately 6.6–17.1% of the isolates presented decreased sensitivity to piperaquine *in vitro*, but no exo-E415G mutation was detected (Robert et al., 2019). Similarly, in a study of 270 *P. falciparum* malaria cases in Mali in 2016, one piperaquine-resistant exo-E415G mutation was found in a patient with no artemisinin resistance genetic background (Diakité et al., 2019). This finding raises concerns about the spread of piperaquine resistance in Africa and other malaria-endemic areas. Epidemiological data related to the piperaquine resistance gene *Pfexo* are scarce globally. In the present study, polymorphisms of *Pfexo* were investigated in *P. falciparum* in returning workers in Wuhan from 2011 to 2019. The results showed that no exo-E415G mutation was detected in the *P. falciparum* malaria samples collected from workers returning from Africa or Southeast Asia; however, three unreported *Pfexo* nonsynonymous mutation sites, D393E, D430Y, and E437K, were detected. Whether these mutations are related to piperaquine resistance needs to be further studied.

Accurate, low-cost, and feasible molecular marker assays are particularly critical for obtaining timely information on *P. falciparum* drug resistance. Currently, the traditional method for detecting *Pfexo* is gene sequencing. However, this approach is impractical for front-line use, especially in remote areas. Traditional AS‒PCR is a commonly used molecular assay that is simple and economical to perform, but a significant disadvantage of this method is the high false positive rate (Ranford-Cartwright et al., 2002; Yang et al., 2017). In this study, AS‒PCR was optimized. First, different artificial mismatches were designed for the antepenultimate base of the AS primers, and the primer with the highest amplification efficiency and specificity was selected. Second, the 3′ end of the primer was modified with phosphorothioate, which improved the ability of the AS primer to distinguish the genotype. Third, the Mg^2+^ concentration, primer concentration, annealing temperature and cycle number were optimized. Finally, the high sensitivity and specificity was demonstrated that the LOD of the AS‒PCR system reached 10^4^ copies/μL and 100 parasites/μL for the clinical samples, with no nonspecific amplification. Additionally, this method is low cost, easy to operate and suitable for primary hospitals and underdeveloped areas. However, AS‒PCR is still time-consuming (more than 2 h per test) and cannot eliminate its dependence on precise thermal control instruments and electrophoresis, which limits its widespread use.

In recent years, with the emergence and outbreak of various emerging infectious diseases worldwide, the POCT strategy, which can rapidly and accurately detect pathogens, has once again become a research hotspot. In particular, various nucleic acid amplification and identification technologies continue to emerge (Song et al., 2021; Xiao et al., 2022). The advent of isothermal amplification techniques has enabled rapid nucleic acid amplification independent of PCR equipment, such as RPA/RAA (Lin et al., 2023), which is highly efficient and simple to perform, compared with traditional PCR. Moreover, the combination of isothermal amplification technology and CRISPR/Cas technology has achieved one step further, and has been successfully applied in the detection of human papillomaviruses, African swine fever, novel coronaviruses, and other pathogens (Bai et al., 2019; Wei et al., 2023; Chen et al., 2018). To eliminate the dependence on PCR equipment, we introduced RAA combined with CRISPR‒Cas12a technology in this study, and the piperaquine resistance-associated gene *Pfexo* genotype was successfully detected. Neither the wild-type nor mutant crRNA could distinguish genotypes when mismatched bases were not introduced, which was considered to be related to the insufficient ability of the crRNA system to identify single-base mutations and the characteristics of the *P. falciparum* gene sequence (high A and T content, more than 80%). Mismatched bases were innovatively introduced into crRNA to improve specificity, although this may reduce detection sensitivity. As a result, the wild-type and mutant crRNA successfully distinguished genotypes with high sensitivity, and the LODs reached 10^3^ copies/μL and 10^4^ copies/μL, respectively. Moreover, two methods for displaying genotype results were designed in the present study to adapt to different use scenarios. The fluorescence curve can be monitored using fluorescence detection equipment, and genotypes can be distinguished according to the curve characteristics. The other method involves directly interpreting the genotype results with the naked eye to determine whether fluorescence appears after irradiation with blue or ultraviolet light. Therefore, RAA‒CRISPR/Cas12a eliminates the tedious agarose gel electrophoresis and visualization process and significantly shortens the detection time to within 1 h. In addition, the RAA amplification reagent is a freeze-dried powder, which is convenient for transportation and storage. However, the costs of RAA amplification reagents, crRNA synthesis, the Cas12 enzyme, and other reagents required by this method are greater than those required for AS‒PCR.

This study has several shortcomings, such as the lack of clinical samples with mutant resistance genes and the inability to detect other unknown gene mutations. In addition, RAA‒CRISPR/Cas12a requires repeated and accurate aspiration and pipetting to add samples during operation, and crRNA is easily degraded, which is a potential challenge when applied to POCT. Nevertheless, isothermal amplification combined with CRISPR technology remains one of the most promising methods for POCT detection. As the method continues to be optimized and improved, these problems will certainly be overcome. For example, the RAA reagent can be mixed with CRISPR/Cas-related reagents to prepare dry powder, so that only samples and water need to be added during detection.

## 5 Conclusion

The study established two rapid detection systems for the *Pfexo* gene: the AS‒PCR assay and the RAA‒CRISPR/Cas12a assay. The minimum detection limit of AS‒PCR is 10^4^ copies/μL, with a low cost and high sensitivity and specificity, and it is suitable for primary hospitals and remote areas. Moreover, the LOD of the RAA‒CRISPR/Cas12a assay was 10^3^ copies/μL, which is more suitable for POCT strategy with more sensitivity, shorter detection time, less equipment dependence. Importantly, we provide more practical detection methods for the early detection of resistance gene mutations related to antimalarial drug resistance, which will contribute to the global malaria elimination program.

## CRediT authorship contribution statement

**Huiyin Zhu:** Writing – original draft, Methodology, Investigation. **Daiqian Zhu:** Writing – original draft, Methodology, Investigation. **Yuting Li:** Methodology, Investigation. **Yun Li:** Methodology, Investigation. **Xiaonan Song:** Methodology, Investigation. **Jinyu Mo:** Formal analysis. **Long Liu:** Methodology, Investigation. **Zhixin Liu:** Methodology, Investigation. **Siqi Wang:** Formal analysis. **Yi Yao:** Formal analysis. **He Yan:** Formal analysis. **Kai Wu:** Resources, Investigation. **Wei Wang:** Writing – review & editing. **Jianhai Yin:** Writing – review & editing, Funding acquisition, Conceptualization. **Min Lin:** Supervision, Conceptualization. **Jian Li:** Writing – review & editing, Supervision, Funding acquisition, Conceptualization.

## Ethics approval and consent to participate

This study was approved by the Medicine Ethical Committee of Hubei University and Wuhan CDC (WHCDCIRB-K-2021013), and informed consent was provided by all participating individuals.

## Consent for publication

Not applicable.

## Availability of data and materials

The datasets generated and/or analyzed during the current study are available from the corresponding author on reasonable request.

## Competing interests

The authors declare that the research was conducted in the absence of any commercial or financial relationships that could be construed as potential conflicts of interest.

## Funding

This study was supported by the Principle Investigator Program of Hubei University of Medicine (HBMUPI202101), the Advantages Discipline Group (Public Health) Project in Higher Education of Hubei Province (2022PHXKQ1), the Open Project of NHC Key Laboratory of Parasite and Vector Biology (NHCKFKT2023-04), and the Three-Year Initiative Plan for Strengthening Public Health System Construction in Shanghai (2023–2025) Principal Investigator Project (GWVI-11.2-XD34).

## Abbreviations

WHO: World Health Organization
pfexo: Plasmodium falciparum exonuclease
RAA: recombinase-aided amplification
AS-PCR: allele-specific PCR
ACTs: artemisinin-based combination therapies
qPCR: quantitative polymerase chain reaction
COVID-19: novel coronavirus disease
POCT: point-of-care testing
LAMP: loop-mediated isothermal amplification
RPA: recombinase polymerase amplification
CRISPR: clustered regularly interspaced short palindromic repeats
SNP: single nucleotide polymorphism
LOD: limit of detection
