# Supplementary figures and images for: Rapid detection of mutations in the suspected piperaquine resistance gene E415G-exo in Plasmodium falciparum exonuclease via AS‒PCR and RAA with CRISPR/Cas12a
Source: Int J Parasitol Drugs Drug Resist. 2024 Oct 28;26:100568. doi: 10.1016/j.ijpddr.2024.100568 (PMC11550206; doi:10.1016/j.ijpddr.2024.100568)

A

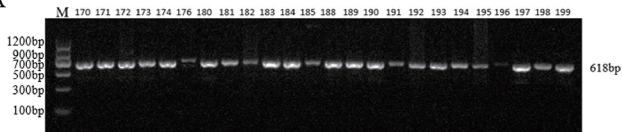

B

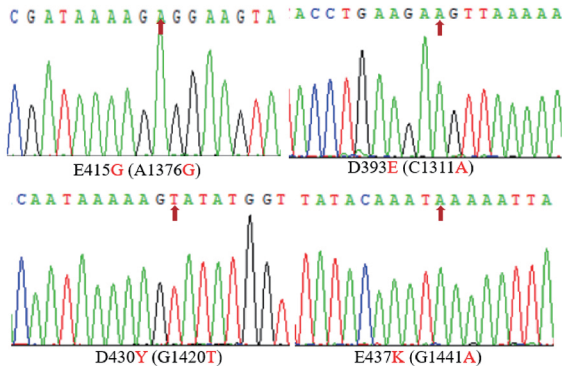

Supplement: Multimedia component 1 [file mmc1.pdf]

**A**

pDNA  
Digested pDNA  
DNA Maker

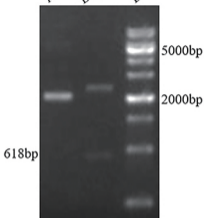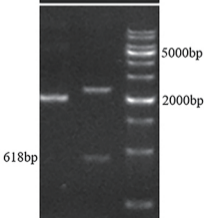

**B**

pUC-*pfexo*(Wild type)

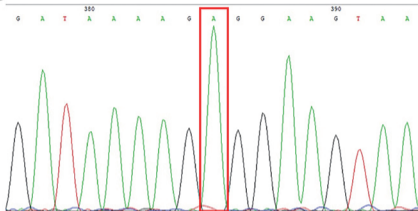

pUC-*pfexo*(Mutant type)

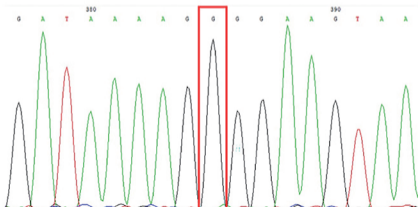

Supplement: Multimedia component 2 [file mmc2.pdf]
